# Risk-stratified pharmacologic strategies for secondary prevention after acute diverticulitis: an exploratory narrative review and research framework

**DOI:** 10.3389/fsurg.2026.1771037

**Published:** 2026-03-10

**Authors:** Michele Schiano di Visconte, Sonia Sarnari, Dario Lo Monaco, Antonio Brillantino, Luigi Marano, Pasquale Talento, Angelo Guttadauro

**Affiliations:** 1 Department of General Surgery, Azienda ULSS 2 “Marca Trevigiana”, Treviso, Italy; 2 Poliambulatori di Treviso, Azienda ULSS 2 “Marca Trevigiana”, Treviso, Italy; 3 General Surgery Resident, Università Degli Studi di Milano-Bicocca, Monza, Italy; 4 Department of General Surgery, A. Cardarelli Hospital, Naples, Italy; 5 Department of Medicine, Academy of Applied Medical and Social Sciences (AMiSNS), Elblag, Poland; 6 Department of General Surgery, Pelvic Floor Center, AULSS-IRCCS Reggio Emilia, Italy; 7 Associate Professor of General Surgery, Università Degli Studi di Milano-Bicocca, Monza, Italy

**Keywords:** DICA score, diverticulitis, mesalazine, probiotics, recurrence, rifaximin, risk stratification

## Abstract

**Background:**

Recurrent acute diverticulitis (RAD) affects up to one-third of patients after the first episode and is associated with impaired quality of life and increased healthcare burden. Although interest in pharmacological prophylaxis is increasing, adequately powered randomized trials have not shown consistent benefits for any agent.

**Aims:**

Synthesize current evidence on pharmacological strategies for secondary prevention of diverticulitis, evaluate the potential of risk stratification and propose an exploratory framework to inform future studies.

**Methods:**

We conducted a structured narrative review in accordance with the SANRA criteria, including randomized controlled trials, observational studies, systematic reviews and international guidelines published between 1995 and 2026. Studies on primary prevention or symptomatic uncomplicated diverticular disease (SUDD) were assessed separately as indirect evidence.

**Results:**

Although subgroup analyses have suggested possible benefits in selected high-risk populations, large randomized trials have not demonstrated a reduction in recurrence with mesalazine or rifaximin. Evidence regarding probiotics remains inconclusive and is largely limited to SUDD. Currently, the major international guidelines do not recommend the use of pharmacological agents for routine secondary prevention. Emerging data indicate that radiologic features, endoscopic severity (DICA score), and biomarker findings, may help identify patients at increased risk of recurrence.

**Conclusions:**

Currently, no pharmacological therapy is available for the universal secondary prevention of diverticulitis. We propose a hypothesis-generating, risk-stratified framework that integrates clinical, endoscopic and radiological markers to support individualized trial design and patient selection. This approach may help guide future randomized studies and refine the preventive strategies.

## 1 Introduction

Recurrent acute diverticulitis (RAD) occurs in 20%–35% of patients after the first episode and represents a relevant burden in terms of morbidity, impaired quality of life and healthcare utilization (1, 2). Historically, elective colectomy has been considered after a limited number of attacks; however, current guidelines increasingly recommend individualized, nonsurgical management (3).

Strategies for preventing diverticulitis can be categorized into three distinct domains (1–3). Primary prevention targets individuals with diverticulosis but no prior episodes, aiming to avoid the first occurrence of diverticulitis in the future. Symptomatic uncomplicated diverticular disease (SUDD) is a separate entity characterized by chronic abdominal symptoms in the absence of acute diverticulitis, with dedicated trials that cannot be directly extrapolated to recurrence prevention. Secondary prevention, which is the exclusive focus of this review, involves strategies to reduce the risk of RAD in patients after a documented first episode (1–3).

Different pharmacologic strategies have been tested for recurrence prevention, including rifaximin, mesalazine and probiotics (4–6); however**,** their efficacy remains controversial. Rifaximin has never been assessed in adequately powered randomized controlled trials for secondary prevention; the only dedicated study (the ROAD trial, NCT03469050) was prematurely interrupted for slow recruitment (4). Moreover, in symptomatic uncomplicated diverticular disease (SUDD), rifaximin failed to show superiority over placebo in a double-blind randomized controlled trial (RCT) (5). However, large phase III RCTs have demonstrated no reduction in recurrence rates compared to placebo (6–8). Conversely, the GISMI trial (9) and the DIVA trial (10) reported a longer recurrence-free interval and improved quality of life in selected patients. These results suggest a potential benefit for specific subgroups, although this has not been confirmed in larger studies.

Evidence for probiotics is even more limited; no randomized controlled trials (RCTs) have specifically evaluated their role in preventing recurrence after acute diverticulitis (AD), and available data are restricted to SUDD (11, 12). Thus, probiotics cannot be considered effective but have only been insufficiently studied in this setting.

The international consensus statements further highlight these limitations. The 2nd International Symposium on Diverticular Disease and the 3rd Symposium concluded that no pharmacological agent is currently recommended for secondary prevention (13, 14).

Nevertheless, risk stratification has emerged as a key element of management. The Diverticular Inflammation and Complication Assessment (DICA) score correlates with recurrence risk (15), and a retrospective multicenter study showed mesalazine (but not rifaximin) could be associated with reduced recurrence in patients with higher DICA categories (16).

Against this background, the present narrative review focuses exclusively on secondary prevention after acute diverticulitis, critically evaluates the available evidence, integrates international consensus, and proposes a risk-stratified framework to inform future research and trial design.

## 2 Methods

This narrative review was conducted in accordance with the SANRA guidelines (17), which provide criteria for assessing the methodological rigor of non-systematic reviews. Given the heterogeneity of the available evidence, a structured narrative approach was used to synthesize and critically assess pharmacological strategies for the secondary prevention of AD.

**Figure 1:**
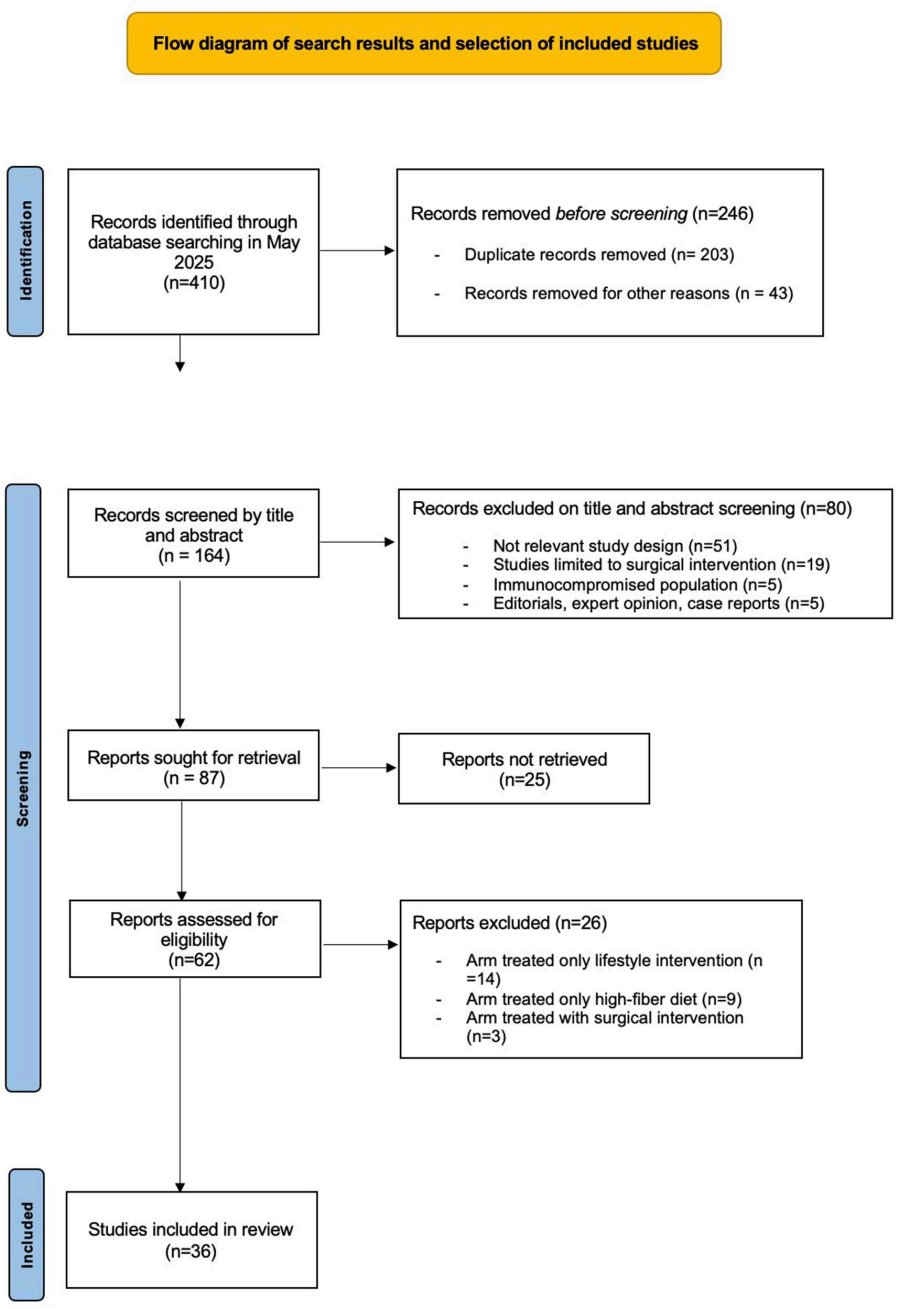
Study selection process for the narrative review.

A systematic literature search was performed across six electronic databases PubMed, Embase, Scopus, Ovid MEDLINE, Web of Science, and Cochrane Central for articles published in English between January 1995 and May 2024. Following peer-review, a targeted updated search was additionally performed to capture recently published international guidelines and *post-hoc* analyses relevant to secondary prevention of acute diverticulitis, including studies published up to January 2026. The search strategy included MeSH terms and free-text keywords related to recurrent diverticulitis, pharmacologic prevention, rifaximin, mesalazine, and probiotics. Additional searches were performed using citation tracking and manual reference screening. The selection process is summarized in Figure 1 (PRISMA-style diagram).

We prespecified a secondary prevention focus: Eligible studies evaluated interventions to prevent the recurrence of diverticulitis in adults after a documented acute episode. Studies on primary prevention and symptomatic uncomplicated diverticular disease (SUDD) were also included when they provided indirect or contextual evidence; for example, when the intervention, mechanism of action, or target population overlapped meaningfully with the setting of secondary prevention. These studies are presented in separate sections and were not pooled with data on recurrence prevention. This inclusion was deemed necessary because of the limited number of high-quality RCTs that specifically target secondary prevention.

Only studies that addressed pharmacological strategies (rifaximin, mesalazine or probiotics) for secondary prevention in adults (aged ≥18 years) were considered. We excluded all the studies that did not report clinical outcomes, focused exclusively on surgical or non-pharmacological interventions, or involved pediatric or immunocompromised populations.

Two independent reviewers extracted the data using a standardized form, including the study design, sample size, population characteristics, interventions, follow-up, and outcomes. Disagreements were resolved through consensus.

Given the substantial heterogeneity across populations, protocols, outcomes, and follow-up durations, a formal meta-analysis was not feasible. Thus, a structured narrative synthesis was adopted, grouping studies by pharmacologic agents and analyzing outcome consistency, methodological quality, and clinical relevance.

The methodological quality of this review was appraised using the SANRA scale (17). Although formal risk-of-bias tools (e.g., ROB 2.0) were not applied, we employed predefined eligibility criteria, dual screening, and consensus discussion to mitigate bias.

## 3 Results

A total of 33 peer-reviewed studies were included in this narrative review, comprising 20 studies (including randomized controlled trials—RCTs, retrospective cohort studies, and systematic reviews) and 13 clinical guidelines. The evidence base showed considerable heterogeneity in terms of the study design, population risk profiles, treatment duration, recurrence definitions, and follow-up timeframes. A summary of the included studies is shown in Table 1; although several trials have explored mesalazine or rifaximin for RAD prevention, the overall quality of evidence remains low to moderate and consistent benefits have not been demonstrated. This underscores the need for better patient selection and stratified study design.

**Table 1:** Summary of peer-reviewed studies evaluating pharmacological strategies for secondary prevention of recurrent acute diverticulitis

Study	Design (Year)	Population	Intervention	Key outcomes	Length follow-up	Outcome	SANRA Score
Bianchi et al. (22)	Meta-analysis (2011)	7 RCTs (*n* = 1,660)	Rifaximin (400 mg bid/day for 7–10 days/month) plus fibre vs. control	Recurrence of diverticulitis	12 months	The pooled RD for complete symptom relief in favour of rifaximin group was 29.0% (95% CI 24.5% to 33.6%; *p* <0.0001)	12
Carabotti et al. (31)	Review (2019)	1,805 patients	5-ASA versus placebo	Prevention of recurrent diverticulitis	NA	No efficacy 5-ASA versus placebo (31.3% vs. 29.8%; RR 0.69, 95% confidence) interval (CI) 0.43–1.09)	10
Carter et al. (6)	Cochrane review (2017)	7 RCTs (*n* = 1,805)	Mesalazine (various doses and regimens) vs. placebo or no treatment	Prevention diverticulitis recurrence	Range 12 to 24 months	Recurrence of diverticulitis: 5-ASA group 31.3%, non-5-ASA group 29.8% (no significant difference)	12
De Bastiani et al. (32)	Retrospective (2021)	286 patients	Rifaximin at a dose of 400 mg bid for 5, 7 or 10 days monthly, up to 3 months.	Diverticulitis recurrence	3 months	Acute diverticulitis occurred in 3.1% patients,	10
Festa et al. (21)	Retrospective (2017)	124 patients	Rifaximin (400 mg bid for 10 days/months) vs. Mesalazine (2.4 g/daily for 10 days/months)	Prevention diverticulitis recurrence	15 months (range 1–50)	Treatment with rifaximin was significantly associated with a reduction in the occurrence of recurrent diverticulitis (HR 0.27; 95% CI 0.10 to 0.72)	10
Gatta et al. (33)	Prospective (2012)	149 patients	Mesalazine 800 mg bid for 10 days/month vs. placebo	Diverticulitis recurrence	60 months	4% recurrence in mesalazine group (2/50) vs. 10.4% in control (8/77); not statistically significant (*p* = 0.1256)	9
Iannone et al. (34)	Systematic review (2018)	13 RCTs (*n* = 3,028)	Mesalazine vs. placebo (various settings)	Disease remission/recurrence acute diverticulitis	More than 12 months	No statistically difference in the likelihood of diverticular disease recurrence between mesalazine and control interventions (9 trials, 2414 partecipants, RR = 0.83, 95% CI = 0.58–1.19)	9
Khan et al. (35)	Meta-analysis (2016)	5 RCTs (*n* = 1,423)	Mesalamine (various dosages and regimens: 800 mg bid, 1.2 g–4.8 g daily, for 12 weeks to 24 months) vs. placebo.	Recurrence of acute diverticulitis	Range 12 months to 104 weeks	No significant difference in recurrence rates between mesalamine and placebo (RR 0.99; 95% CI: 0.74–1.34; *p* = 0.96)	12
Khan et al. (36)	Meta-analysis (2018)	6 RCTs (*n* = 1918)	Mesalazine 2.4 g/day vs. placebo	Recurrence of diverticulitis		No difference in the recurrence of diverticulitis between the mesalazine and placebo (OR 1.20, 95% CI 0.96–1.50, *p* = 0.11)	12
Koch et al. (3)	Systematic review & meta-analysis (2023)	8 RCTs (*n* = 3,013)	Rifaximin (400 mg bid for 7–10 days/months vs. standard dietary fiber supplement	Prevention diverticulitis	Median 42 months (range: 6–84)	Secondary prevention: the risk difference was statistically significant in favor of rifaximin (−0,24, or −24%, CI −47 to −2%).	11
Kruis et al. (25)	RCT (2017)	645 patients	Mesalazine (1.5 g/daily) vs. placebo for 48 weeks/mesalazine (3.0 g/daily) vs. placebo for 96 weeks/	Preventing diverticulitis recurrence,	12–24 months	In SAG- 37 the proportion of recurrence-free patients during 48 weeks was 67.9% with mesalazine and 74.4% with placebo (*p* = .226)/In SAG-51 The proportion of recurrence-free patients over 48 weeks was 46.0% with 1.5 g mesalazine, 52.0% with 3 g mesalazine and 58.0% with placebo (*p* = .860)	11
Lamiki et al. (12)	Prospective (2010)	46 patients	SCM-III symbiotic mixture (L. acidophilus 145, L. helveticus ATC 15009, Bifidobacterium spp. 420, phytoextracts); 10 ml TID	Preventing diverticulitis recurrence	6 months	68.8% remained symptom-free at 6 months	11
Lanas et al. (20)	Prospective (2013)	165 patients	Rifamixin 400 mg bid/plus fibre 7 g/day for 7 days vs. fibre 7 g/day for 7 days each month	Diverticulitis recurrence	12 months	Rifamixine/fibre 10.4 diverticulitis recurrence vs. fibre alone 19,3% (OR: 3.20; *p* = 0.025)	10
Parente et al. (9)	RCT (2013)	92 patients	Mesalazine 800 mg bid for 10 days/month vs. placebo, for 24 months	Prevention of diverticulitis recurrence/Improvement quality of life	24 months	Recurrence at 24 months: 13.3% in mesalazine group vs. 27.7% in placebo; RR not statistically significant (*p* = 0.1011)	11
Picchio et al. (27)	Systematic review & meta-analysis (2016)	6 RCTs (*n* = 1021)	Mesalazine (various doses and regimens) vs. Placebo	Prevention of diverticulitis occurrence	Range 1 to 48 months	The percentage of absolute risk reduction was significant when compared to placebo	11
Raskin et al. (7)	RCT (2014)	1184 patients (590 patients in PREVENT 1 and 592 patients in PREVENT 2)	Mesalazine (doses ranging 1.6–4.8 g/day) vs. placebo for 12 weeks	Prevention diverticulitis recurrence	24 months	No statistically significant difference in recurrence of symptoms between mesalazine and placebo groups; symptom relief seen in both arms. PREVENT 1 (53–63% vs. 65%, *p* = n.s.) and in PREVENT 2 (59–69% vs. 68%, *p* = n.s.)	9
Stefanelli et al. (26)	Review (2020)	12 Studies (RCTs, open trial, retrospective)	Mesalazine 2.4 g/day for 10 days/month vs. no treatment	Prevention of first episode diverticulitis/ preventing diverticulitis recurrence	NA	The role of 5-ASA in the prevention of diverticulitis recurrence is still uncertain	11
Stollman et al. (10)	RCT (2013)	92 patiens	Mesalazine 2.4 g/day vs. mesalazine 2.4 g/day +bifidobacterium infantis vs. placebo	Prevention diverticulitis recurrence	12 months	Responders remained consistently higher in the mesalazine group (week 26, 66.7%; week 39, 64.3%; week 52, 66.7%) compared with combined mesalamine/probiotic group (week 26, 42.3%; week 39, 37.5%; week 52, 29.2%) or the placebo group (week 26, 47.8%; week 39, 45.5%; week 52, 50%)	10
Tursi et al. (11)	RCT (2013)	210 patients	Group M. mesalazine 800 mg bid/day for 10 days/month vs. placebo. Group L Lactobacillus casei DG (24 billion CFU/day) for 10 days/month vs. placebo; Group LM mesalazine 800 mg BID for 10 days/month plus Lactobacillus casei DG (24 billion CFU/day) for 10 days/month; Group P: placebo day for 10 days/month;	Prevention diverticulitis recurrence	12 months	Mesalazine, alone or in combination, had lower diverticulitisoccurrence (0% and 1.81% vs. 12%, *p* = 0.003)	10
Urushidani et al. (8)	Systematic review & meta-analysis (2017)	8 RCTs	5-aminosalicylic acid (5-ASA) agents vs. placebo or no treatment	Prevention diverticulitis recurrence	Range 6–24 months,	5 -ASA agents were not superior to placebo in preventing recurrent diverticulitis (RR 0.86, 95% CI 0.63 to 1.17, I2 =60%)	11

### 3.1 Rifaximin-based regimens

Rifaximin has been investigated in diverticular disease, generally in cyclic regimens (400–800 mg bid for 7–10 days per month) in combination with dietary fiber (18). Most of the available evidence comes from observational cohorts or studies of SUDD rather than adequately powered secondary-prevention RCTs. No adequately powered RCTs have tested rifaximin for true secondary prevention. Most evidence is derived from observational cohorts or SUDD studies and should be interpreted as indirect rather than definitive proof. In these indirect settings, lower recurrence or improved symptom control has been reported. For example, Banasiewicz et al. observed 28% recurrence compared to 60% in controls (*p* < 0.0001) (19), and Lanas et al. reported 10.4% vs. 19.3% recurrence with fiber alone (*p* = 0.033) (20). Festa et al. noted a 9.7% recurrence rate with rifaximin vs. 26.9% with mesalazine (21), while another cohort study found better outcomes with rifaximin–mesalazine combination (11.4%) than with rifaximin alone (32.2%).

Systematic reviews have explored the use of this approach. Koch et al. reported an absolute risk reduction of 24% (95% CI: −47% to −2%), although high heterogeneity (*I*^2^ = 92%) limits the reliability of the pooled estimates (3). Bianchi et al. found that 64% of patients receiving rifaximin plus fiber remained symptom-free at one year compared to 34.9% receiving fiber alone (22). Stallinger et al. reported that ≥90% of patients treated for three months had minimal or no symptoms, with a low adverse event rate (0.6%) (23). Zullo et al. reviewed four RCTs and estimated a decrease in first-episode incidence from 2.75% to 1.03%, however these were open-label, short-term trials that focused mainly on primary prevention or SUDD (24).

The only dedicated multicenter RCT for secondary prevention, the ROAD trial, was terminated early owing to slow recruitment, leaving it underpowered. No significant differences were detected between rifaximin and placebo regarding recurrence rates or diverticular complications over 12 months. However, rifaximin improved abdominal pain, bowel habits, and quality of life, particularly at higher doses, and demonstrated a favorable safety profile (4). Taken together, evidence from indirect studies suggests a potential benefit, but current data are insufficient to recommend routine rifaximin for the secondary prevention of AD (4).

### 3.2 Mesalazine

Evidence for mesalazine in preventing recurrent diverticulitis remains inconsistent (6). Several large randomized controlled trials (RCTs) failed to demonstrate a reduction in recurrence: the SAG-37 and SAG-51 trials (675 patients) showed no benefit over placebo (25), while the PREVENT-1 and PREVENT-2 phase III trials (>1,100 patients) confirmed no meaningful difference in recurrence-free survival over two years (7). A Cochrane meta-analysis of seven RCTs including 1,805 patients similarly reported comparable recurrence rates with mesalazine (33.1%) and placebo (29.8%) (6). Consistently, Urushidani et al. found no advantage of 5-aminosalicylic acid over control interventions (RR: 0.86; 95% CI: 0.63–1.17) (8), and Stefanelli et al. reported no preventive benefit in most of the 12 studies analyzed (26).

Smaller studies and subgroup analyses have suggested possible effects in selected populations. Picchio et al. pooled two small RCTs and observed lower recurrence rates with mesalazine compared with placebo (19.3% vs. 33.3%; OR: 0.35; 95% CI: 0.17–0.70; *p* = 0.003), although overall study quality was limited (27). Calini et al. reported symptomatic improvement without achievement of remission (28). In the GISMI trial, 96 patients with a first episode of uncomplicated diverticulitis were randomized to intermittent mesalazine or placebo for 24 months; at two years, recurrence occurred in 13.3% vs. 27.7%, respectively (RR: 0.49; *p* = 0.10). Although this difference did not reach statistical significance, mesalazine was associated with significant improvements in physical condition and quality-of-life scores (*p* = 0.02) and with a 20% reduction in concomitant gastrointestinal drug use (9).

Similarly, the DIVA trial showed no significant difference in recurrence rates (28% vs. 31%); however, mesalazine was associated with higher rates of complete symptom resolution at weeks 6 and 52 and with greater improvement in rectosigmoid symptoms compared with placebo (10). Collectively, these “gray” findings are consistent with an underpowered signal rather than definitive efficacy. In GISMI, the recurrence reduction (13.3% vs. 27.7%) did not reach statistical significance (*p* = 0.10), plausibly because of the small sample size, yet mesalazine was associated with clinically meaningful improvements in quality of life and reduced concomitant medication use (9). Similarly, in DIVA, the lack of a statistically significant difference in recurrence may have been influenced by the study structure (3 months of therapy followed by 9 months of observation), despite a longer mean time to first recurrence and significantly higher complete symptomatic response, particularly for rectosigmoid symptoms, at weeks 6 and 52 vs. placebo (10).

Overall, large phase III trials did not demonstrate a consistent reduction in objectively defined recurrence in unselected populations; however, smaller trials suggest possible symptomatic and quality-of-life benefits and a potential signal in selected subgroups. A multicenter retrospective study also suggested that mesalazine, but not rifaximin, is associated with reduced recurrence in patients with higher DICA categories (16), supporting the potential role of endoscopic stratification. These observations remain hypothesis-generating, and mesalazine is not recommended for routine secondary prevention.

### 3.3 Probiotics

No randomized controlled trial has directly evaluated probiotics for secondary prevention of AD. Although no randomized trials have evaluated probiotics specifically for secondary prevention after an index episode of acute diverticulitis, a very recent *post-hoc* analysis provides indirect evidence from a related clinical setting. In a 12-month, multicenter, double-blind, placebo-controlled trial conducted in patients with symptomatic uncomplicated diverticular disease (SUDD) without prior diverticulitis, cyclic administration of Lactobacillus paracasei CNCM I 1572 (24 billion CFU/day for 10 days per month) was associated with a significantly lower incidence of first, CT-confirmed acute diverticulitis compared with placebo (1.8% vs. 12%; *p* = 0.036) (29). While these findings cannot be extrapolated to post-diverticulitis populations, they support the mechanistic rationale for microbiota-targeted interventions and underscore the need for adequately powered trials testing defined strains in true secondary prevention after acute diverticulitis.

Most evidence is derived from SUDD cohorts, which limits their applicability to true post-AD populations. For instance, Tursi et al. tested Lactobacillus casei DG, alone or combined with mesalazine, reporting improvements in abdominal pain and bowel function and, in some analyses, fewer symptomatic relapses, but recurrence was not a predefined endpoint, and the enrolled patients had SUDD rather than prior AD (11). Another open-label prospective study of L. acidophilus and Bifidobacterium spp. found that 68% of patients remained symptom-free at six months, and over 78% rated treatment as effective (12), again in SUDD rather than post-AD settings.

Systematic reviews, including the Cochrane analysis by Carter et al., found no statistically significant benefit, citing heterogeneity in probiotic strains, dosages, regimens, and outcome definitions (6). In summary, the evidence for the use of probiotics to prevent recurrent diverticulitis remains insufficient. No RCT has addressed this question in true secondary prevention, and the available data are restricted to SUDD, precluding any firm recommendation for clinical practice.

These findings are consistent with the international consensus (2nd and 3rd International Symposia), which concluded that no pharmacological agent, including probiotics, can be recommended for the secondary prevention of diverticulitis (13, 14).

## 4 Discussion

The available evidence on pharmacological strategies for secondary prevention of AD is constrained by several methodological shortcomings. First, there was substantial heterogeneity in study design, patient selection, dosing regimens and outcome definitions. Second, many studies had small sample sizes and were conducted with open-label or single-center designs, thus limiting external validity. Third, recurrence was not consistently defined as the primary endpoint, particularly in trials extrapolated from SUDD or other indirect populations. Collectively, these limitations preclude a robust meta-analysis and necessitate a cautious narrative synthesis (3, 6, 8, 25). Prior position papers, including the Italian SIGE/SICCR guidelines (30), emphasized the same barriers, heterogeneity, and underpowered trials and concluded that no recommendation can currently be made for routine rifaximin use in this setting. Moreover, large phase *III* randomized controlled trials (PREVENT 1 and 2, SAG-37, SAG-51) that prespecified CT-confirmed recurrence as the primary endpoint consistently failed to demonstrate any benefit of mesalazine over placebo (6–8, 25, 31–36) These findings underscore the discrepancy between exploratory signals from smaller studies and the negative results of adequately powered, high-quality trials.

### 4.1 Clinical rationale and comparative effectiveness

**Figure 2:**
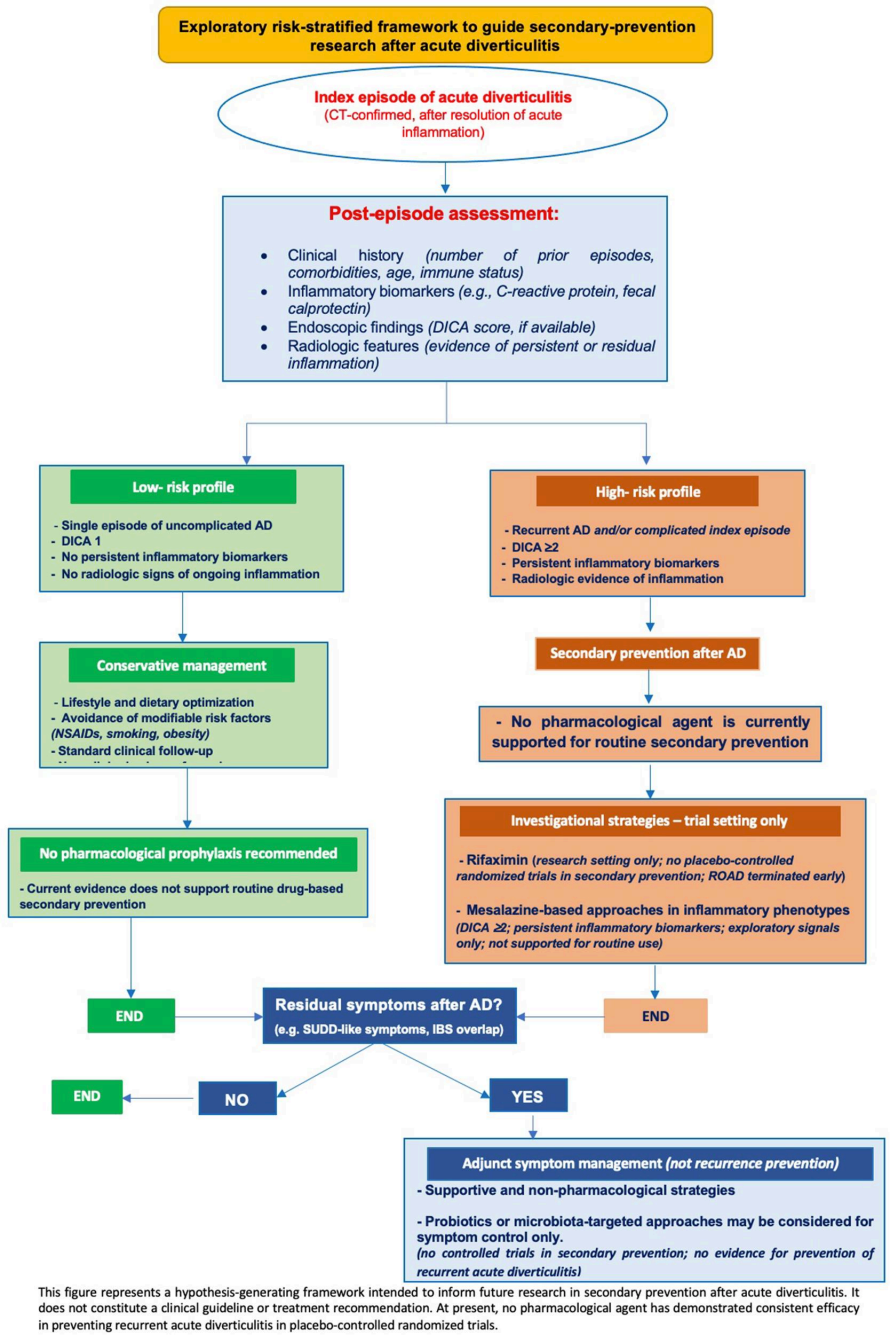
Exploratory risk-stratified framework to inform secondary-prevention research after acute diverticulitis.

Rifaximin is a non-absorbable antibiotic with antimicrobial and anti-inflammatory activities. Most of the available evidence is derived from observational cohorts or from studies conducted in SUDD or primary prevention settings; consequently, existing meta-analyses remain heterogeneous and indirect, precluding firm conclusions (37–39). While long-term clinical experience with other indications confirms its favorable safety and tolerability profile (40, 41), these data do not establish efficacy for secondary prevention after AD. Supporting evidence is further constrained by small sample sizes, heterogeneous study designs and variability in dosing protocols (2). The only randomized controlled trial evaluating rifaximin for the primary prevention of AD in patients with SUDD failed to demonstrate superiority over placebo in reducing diverticulitis incidence, further limiting the strength of the evidence (5). Moreover, the absence of large multicenter RCTs and unresolved cost-effectiveness concerns have prevented rifaximin from gaining universal endorsement in international guidelines. Nonetheless, real-world data suggest that the drug continues to be widely used in parts of Europe, with approximately 43% of surveyed clinicians reporting its prescription for recurrence prevention (42). In the conceptual framework proposed in this review (Figure 2), rifaximin alone is presented as an investigational option to be evaluated in future stratified trials involving high-risk patients.

Mesalazine, a 5-aminosalicylic acid compound widely used in inflammatory bowel disease, has been hypothesized to reduce diverticulitis recurrence by attenuating mucosal inflammation (8, 15). However, multiple randomized controlled trials have not demonstrated a statistically significant preventive effect on recurrence in unselected secondary-prevention populations. In particular, large phase III programs (PREVENT 1–2, SAG-37, and SAG-51) consistently reported no difference in recurrence-free survival compared to placebo (6–8, 25). Notably, these large programs predominantly enrolled patients with more established recurrent disease (often ≥2 prior episodes), which may have diluted any treatment effect if mesalazine were to be more effective earlier in the disease course. Such enrollment criteria may have reduced the ability to detect a preventive effect if mesalazine were more effective earlier in the disease course, after a first episode. This hypothesis is indirectly supported by the GISMI population (single prior episode) and by subgroup signals reported in the SAG program among patients with only one episode before enrollment; nevertheless, these observations remain exploratory and require prospective confirmation. Meta-analyses confirm these findings; however, several of these pooled studies were conducted in heterogeneous settings (including SUDD, primary prevention and secondary prevention), which limits the interpretability of their estimates when applied to true post-AD populations.

Smaller trials and subgroup analyses have suggested possible benefits of SUDD, especially when mesalazine was combined with probiotics; however, these effects were limited to symptom improvement and did not translate into reduced recurrence risk (27, 28). A retrospective multicenter study further suggested that mesalazine, but not rifaximin, was associated with lower recurrence rates in patients with higher DICA categories, supporting the potential role of endoscopic stratification, although these findings remain exploratory (15, 43).

An isolated study investigating high-dose mesalazine (>4 g/day) reported a statistically significant reduction in recurrence at 4.8 g/day vs. placebo (7). However, this apparent effect was largely driven by treatment discontinuations classified as failures, and the trial was judged to have a high risk of bias in the NICE assessment (44). Thus, any potential dose-dependent efficacy remains hypothetical and is not supported by robust evidence (26).

Additional exploratory data were derived from small or underpowered trials that reported mixed results. Although not meeting their primary endpoints, the GISMI and DIVA trials observed a possible prolongation of recurrence-free intervals and improved patient-reported outcomes among patients treated with mesalazine (9, 10). Moreover, a *post hoc* analysis by Tursi et al. found that mesalazine was associated with lower recurrence rates in patients with endoscopic evidence of severe disease (DICA ≥ 2), suggesting that anti-inflammatory strategies may hold greater potential in high-risk subgroups (16). While these findings remain hypothesis-generating, they support the rationale for incorporating endoscopic and clinical stratification into future trial designs.

Overall, mesalazine is generally well tolerated, with uncommon adverse events including nephrotoxicity, hepatotoxicity, pancreatitis, cardiotoxicity, hypersensitivity, musculoskeletal complaints and rare sexual or respiratory effects (26).

Probiotics. There is currently insufficient evidence to support the routine use of probiotics for secondary prevention after acute diverticulitis. Reported benefits are derived almost exclusively from studies on SUDD, where outcomes focused on symptom relief or reductions in symptomatic relapses, rather than on objectively defined recurrence of diverticulitis (11, 12). From a translational standpoint, the strongest contemporary signal for probiotic prophylaxis comes from primary-prevention data in SUDD rather than from post-diverticulitis cohorts. In particular, a recent *post-hoc* analysis of a randomized, double-blind, placebo-controlled trial reported that cyclic Lactobacillus paracasei CNCM I 1572 reduced the occurrence of first, CT-confirmed acute diverticulitis over 12 months compared with placebo (1.8% vs. 12%; *p* = 0.036) (29). These results suggest that strain-specific microbiome modulation may influence diverticulitis pathobiology and provide a biologically plausible rationale to investigate probiotics in secondary prevention; however, they remain indirect and should not be interpreted as evidence of efficacy after an index episode of acute diverticulitis. Accordingly, probiotics should be considered investigational for secondary prevention until confirmed in dedicated, adequately powered RCTs enrolling post-AD patients with standardized recurrence definitions and objective confirmation. Among these, a double-blind randomized trial by Tursi et al. investigated Lactobacillus casei DG, alone or in combination with mesalazine, reporting symptomatic improvement in SUDD but no significant impact on the recurrence of acute diverticulitis (11). This rationale is further supported by recent evidence showing that L. paracasei CNCM I 1572 is superior to placebo in preventing diverticulitis occurrence by modulating gut microbiota and reducing low-grade inflammation, supporting a biological rationale to test defined probiotic strains in future secondary-prevention randomized controlled trials (29). No RCT has specifically evaluated probiotics in post-AD populations. Systematic reviews have consistently highlighted the absence of reproducible benefits, further emphasizing the heterogeneity of probiotic strains, dosages, treatment regimens, and outcome definitions (6). Therefore, probiotics cannot currently be recommended as standalone agents for preventing recurrence after acute diverticulitis.

Across all pharmacological agents examined, the available evidence does not support routine prophylaxis to prevent recurrent diverticulitis. Apparent signals of benefit, when present, originate from small, indirect, or observational studies and should be regarded as hypothesis-generating only, pending confirmation in adequately powered, stratified randomized controlled trials. In this context, we propose an exploratory conceptual framework (Figure 2) to guide future research by integrating the endoscopic, radiological, and clinical risk factors. Such an approach may help to identify subgroups of patients that are more likely to benefit from targeted preventive strategies and provide a structured rationale for trial design in secondary prevention.

### 4.2 Clinical guidelines stance and international divergences

Most international guidelines do not recommend routine pharmacological prophylaxis with rifaximin, mesalazine, or probiotics for preventing recurrent diverticulitis. In this context, the Global guidelines on diverticular disease of the colon (Fiesole Consensus report), recently published in Gut in 2025, provide a comprehensive, internationally endorsed, GRADE-based framework addressing definitions, diagnostic pathways, medical management, and surgical indications for diverticular disease. With specific regard to secondary prevention after an index episode of acute diverticulitis, the Consensus delivers a strong recommendation supported by high-quality evidence stating that there is no evidence supporting the efficacy of rifaximin and/or mesalazine for either primary or secondary prevention of acute diverticulitis (45). Beyond pharmacological considerations, the document underscores that preventive strategies should primarily focus on modifiable risk factors, including adoption of a high-fibre diet, weight management, and smoking cessation, alongside a careful medication review, recommending avoidance or minimization of NSAIDs, systemic corticosteroids, and opioids whenever feasible. Furthermore, the Consensus emphasizes the importance of structured post-episode follow-up, including colon evaluation by colonoscopy or CT colonography within the first year when not recently performed, to exclude underlying malignancy and to reassess disease course. Collectively, these recommendations further reinforce the conservative stance adopted by most contemporary guidelines against routine pharmacological prophylaxis for recurrent diverticulitis, while clearly identifying lifestyle optimization and appropriate follow-up as the evidence-based pillars of secondary prevention (45).

The American Gastroenterological Association (AGA) (2, 46) and the American College of Physicians (ACP) (47) emphasize lifestyle-based strategies (diet, weight management, smoking cessation, physical activity) and do not support pharmacologic interventions. Similarly, the European Society of Coloproctology (ESCP) (48), UK National Institute for Health and Care Excellence (NICE) (44), and German consensus guidelines (49, 50) advise against mesalazine, rifaximin, or probiotics, citing limited evidence and potential for overtreatment. Updated Italian guidelines explicitly discourage their use (30), whereas Korean and Japanese societies do not endorse mesalazine or probiotics in this context (51, 52).

By contrast, a few national guidelines take a more permissive stance. The Dutch (53) and Danish (54) recommendations acknowledge rifaximin as a potential option for selected patients with recurrent disease. Dutch guidelines also considers mesalazine or probiotics when alternative diagnoses are excluded. Polish guidelines cautiously support rifaximin but do not recommend mesalazine, and the role of probiotics remains unclear (55). The now outdated SICCR position endorses rifaximin (56), whereas the World Gastroenterology Organisation (WGO) has not issued a clear recommendation (57). Furthermore, the recent Global Guidelines (Fiesole Consensus, 2025) reinforce this cautious stance, stating that pharmacological secondary prevention should not be routinely recommended for all patients, but rather tailored based on individual risk factors and the severity of the primary episode (45). A comparative overview of the pharmacological recommendations across the major guidelines is presented in Table 2.

**Table 2:** Summary of international guideline recommendations on pharmacologic prophylaxis for recurrent acute diverticulitis.

Study	Guideline	Rifaximin	Mesalazine	Probiotics	Strength of recommendation	Quality of evidence
Peery et al. (10), Stollman et al. (46)	AGA^a^	Not recommended	Not recommended	Not recommended	Conditional	Very low
Qaseem et al. (47)	ACP^b^	Not recommended	Not recommended	Not recommended	Conditional	Very low
Schultz et al. (48)	ESCP^c^	Not recommended	Not recommended	Not recommended	Conditional	NA
NICE^d^ (44)	NICE^d^	Not recommended	Not recommended	Not recommended	Conditional	Very low
Kruis et al. (49)	German	Not recommended	Not recommended	Not recommended	Conditional	Very low
Binda et al. (56)	SICCR^e^	Positive*	Not recommended	Unclear**	NA^i^	NA^i^
Nagata et al (51)	JGA^f^	Positive*	Not recommended	Unclear**	Conditional	Very low
Murphy et al. (57)	WGO^g^	Unclear**	Unclear**	Unclear**	NA^i^	NA^i^
Carabotti et al. (30)	Italian	Not recommended	Not recommended	Not recommended	Conditional	Very low
Lee et al. (52)	Korean	Not recommended	Not recommended	Unclear**	NA^i^	NA^i^
Andeweg et al. (53)	Dutch	Positive*	Positive*	Positive*	Recommendation	Moderate
Andersen et al. (54)	Danish	Positive*	Not recommended	NA^i^	NA^i^	Very Low
Pietrzack et al. (55)	Polish	Positive*	Not recommended	Absent	NA^i^	NA^i^
Tursi et al. (45)	Italian^h^	Not recommended	Not recommended	Not recommended	Strong	High

^a^ American gastroenterological association.

^b^ American college of physician.

^c^ European society of coloproctology.

^d^ National institute for health and care excellence.

^e^ Società Italiana di chirurgia colo-rettale.

^f^ Japan gastroenterological association.

^g^ World gastroenterology organisation.

^h^ Fiesole consensus: global guidelines on diverticular disease of the colon.

^i^ NA, Not applicable or not assessed

* Positive = The guideline supports or recommends the intervention for prophylaxis.

** Unclear = The guideline supports or recommends the intervention for prophylaxis.

### 4.3 Integration of imaging and endoscopy in preventing diverticulitis recurrence

Radiological and endoscopic modalities are valuable tools for risk stratification and inform individualized management of acute diverticulitis. Computed tomography (CT) remains the standard for diagnosing acute episodes and may reveal the risk of recurrence through baseline inflammatory and morphological markers. In a large retrospective cohort study, Dickerson et al. identified maximum colonic wall thickness and subjective inflammation severity on index CT as independent predictors of recurrence, with hazard ratios of 1.07 and 1.36, respectively (*p* < 0.001 and *p* = 0.003, respectively) (58). These parameters yielded a one-year recurrence risk ranging from 6% to 33% (58). These findings underscore the utility of CT beyond acute diagnostics as a decision support tool when considering elective surgical strategies.

In the post-acute setting, endoscopic assessment further refines the risk stratification. The DICA score proposed by Tursi et al. uses endoscopic features (including the extent of diverticulosis, mucosal hyperemia, edema, erosions, and segmental involvement) to classify patients from DICA 1 (low-risk) to DICA 3 (high-risk) (16). Subsequent validation demonstrated a significant correlation between higher DICA classes and increased recurrence and complication rates (23.7% for DICA class 2 and 48.5% for DICA class 3) over a 24-month period (16, 43). Recently, the DICA International Group reported a *post-hoc* analysis of a large prospective, multicenter cohort in which baseline medical strategies were evaluated according to endoscopic severity (DICA) to prevent incident acute diverticulitis. In this real-world, non-randomized setting (*n* = 1,945), risk-adjusted analyses suggested that mesalazine was associated with a lower hazard of acute diverticulitis compared with rifaximin and mesalazine–rifaximin combination therapy, with the most consistent signal observed in DICA 2 patients. Although this evidence pertains to primary prevention (diverticulosis without prior diverticulitis) and cannot be directly extrapolated to post–acute diverticulitis populations, it supports the conceptual premise that endoscopic risk stratification (DICA, and related composite scores such as CODA) may be leveraged to design and test individualized prophylactic strategies in secondary prevention trials, focusing on higher-risk subgroups (59). These findings support the role of colonoscopy in excluding malignancy and predicting the disease course. While the DICA stratifies risk (23.7% in DICA 2 and 48.5% in DICA 3 at 24 months) (16, 43), it has not been validated to guide pharmacologic prophylaxis and should primarily be used to select candidates for closer follow-up or enrollment in stratified trials.

Radiological and endoscopic assessments provide complementary information CT captures acute-phase inflammation and complications, whereas endoscopy characterizes chronic mucosal diseases. An integrated CT—Endoscopy approach may inform individualized strategies, helping to prioritize closer surveillance and timely surgical consultation; any pharmacological prophylaxis should be considered only in clinical trials.

### 4.4 Economic considerations

The long-term economic impact of pharmacological strategies to prevent RAD requires a thorough evaluation. Among the available agents, rifaximin is widely prescribed in some settings despite limited evidence of its efficacy. Despite high drug costs and variable reimbursements, their use remains widespread in some European contexts (24, 56). Based on model assumptions, Tursi et al. estimated a per-patient cost of approximately €14,226 for cyclic rifaximin (400 mg bid for 7–10 days/month), with projected national expenditure exceeding €300 million if broadly adopted (43).

Although mesalazine and probiotics are less expensive, their lack of proven efficacy raises concerns about inefficient resource utilization. Therefore, cost-effectiveness analyses should consider not only drug acquisition costs but also downstream consequences, including hospitalization, lost productivity, and quality of life.

Patient preference also plays a key role, and many individuals favor proactive prevention despite the conservative stance of most guidelines.

Future analyses should adopt risk-stratified, payer-relevant perspectives and incorporate drug costs, hospitalizations, productivity loss, and health-related quality of life metrics (43). Given the absence of demonstrable efficacy in adequately powered randomized controlled trials, formal cost-effectiveness evaluations remain speculative, and priority should be given to trial-based economic analyses.

### 4.5 Integrated framework for decision-making

Pharmacological strategies for the prevention of recurrent acute diverticulitis (RAD) should ideally be embedded within a structured, risk-adapted framework integrating available clinical evidence, patient-specific characteristics, and real-world feasibility. Current international guidelines remain cautious, reflecting inconsistent and largely negative results from randomized trials. While exploratory signals have been reported in selected clinical contexts, such as patients with multiple prior recurrences, low-grade inflammatory activity, or SUDD, these findings remain unconfirmed and should be interpreted with caution (50–53, 60).

A risk-stratified conceptual approach has been increasingly proposed, incorporating variables associated with a higher likelihood of recurrence, including:

– History of two or more prior episodes
– Elevated inflammatory biomarkers (e.g., C-reactive protein, fecal calprotectin)
– Endoscopic severity (DICA score ≥2)
– Radiologic evidence of chronic or residual inflammation
– Age <50 or high recurrence risk based on prior events, immunocompromised patients

Although these parameters may assist in identifying patients at higher risk, they have not been prospectively validated to guide pharmacological secondary prevention. In particular, while the DICA classification has demonstrated prognostic value for recurrence risk, its role in selecting patients for preventive pharmacological strategies remains to be established in stratified, prospective trials (16, 48). The integration of clinical, endoscopic, radiologic, and biomarker-based information may improve risk assessment, but does not currently justify treatment allocation.

Several pharmacological approaches have been discussed in the literature within this risk-based conceptual framework. Cyclic rifaximin regimens (e.g., 400 mg twice daily for 7 days per month for 6–12 months), typically combined with dietary fiber, have been proposed; however, such strategies remain hypothesis-generating and are not supported by adequately powered randomized controlled trials in the setting of secondary prevention (60, 61). Similarly, probiotic supplementation has been explored primarily for the management of post-diverticulitis functional symptoms, but no evidence supports its use in preventing recurrent acute diverticulitis (6, 11, 12).

Conversely, in low-risk patients, such as those experiencing a single uncomplicated episode without persistent symptoms, current evidence does not support routine pharmacological prophylaxis. In these cases, dietary optimization, lifestyle modification, and standard clinical follow-up remain the recommended management strategies (2, 44, 47, 48).

Accordingly, any structured framework for risk-adapted prevention must be interpreted with caution. Conceptual tools such as algorithms or treatment schemas may be useful to illustrate individualized research hypotheses; however, the proposed framework shown in Figure 2 should be regarded strictly as an exploratory, hypothesis-generating model. Its clinical applicability and predictive value require confirmation in prospective, stratified, randomized trials before any consideration for routine clinical use (28, 53, 54).

### 4.6 Limitations of the present review

This narrative review had several limitations that warrant consideration. First, the inclusion of diverse study designs, including RCTs, observational cohorts and both systematic and narrative reviews, introduces potential selection and reporting bias. Second, the marked heterogeneity in patient populations, interventions, outcome definitions, and follow-up durations precluded the performance of a formal meta-analysis. In addition, several systematic reviews and meta-analyses have pooled studies conducted in heterogeneous clinical settings, including SUDD, primary prevention, and true secondary prevention after acute diverticulitis, thereby limiting the interpretability of aggregated estimates when applied specifically to secondary prevention (8, 33–35). Third, no standardized risk-of-bias tool (e.g., ROB 2.0) was applied to evaluate the internal validity of the included RCTs. Fourth, the absence of patient-level data prevented subgroup analyses based on clinically relevant factors such as age, biomarkers, or DICA classification.

Moreover, recurrence was frequently not prespecified as the primary endpoint, and many trials were underpowered, with small sample sizes and limited follow-up durations. Narrative reviews are generally consistent in their content and adequately referenced; however, they often lack structured search strategies or formal bias assessments. When appraised using the SANRA checklist, methodological quality ranged from moderate to high (scores 9–13) (17).

Finally, the overall body of evidence is constrained by the lack of adequately powered, head-to-head randomized trials comparing pharmacological strategies, the underrepresentation of high-risk or multimorbid patients, and the inconsistent reporting of key outcomes. These limitations collectively underscore the need for large, prospective, stratified clinical trials with harmonized definitions and extended follow-up to yield more robust and generalizable conclusions.

### 4.7 Future research directions

Addressing the current gaps in the pharmacological prevention of RAD requires rigorous, stratified trials in well-defined patient subgroups. The key priorities for future research are as follows:

#### 4.7.1 *Stratified randomized controlled trials (RCTs)

Prospective trials should enroll patients according to validated risk markers such as DICA score, CRP, fecal calprotectin, and imaging evidence of residual inflammation to better identify those who may benefit from prophylaxis.

#### 4.7.2 *Standardized endpoints

Future studies should adopt harmonized primary outcomes, including recurrence-free survival at 12 and 24 months, CT-confirmed episodes, and the time to first recurrence. The secondary outcomes included symptom burden, adverse events, and health-related quality of life.

#### 4.7.3 *Patient-reported outcome measures (PROMs)

Validated tools (e.g., GIQLI and PROMIS-GI) should be integrated as secondary or co-primary endpoints to capture symptom control and functional status.

#### 4.7.4 *Head-to-head comparisons

Direct comparisons between rifaximin, mesalazine, and probiotics, alone or in combination, are required to clarify their relative efficacy, tolerability, and cost-effectiveness in stratified cohorts.

#### 4.7.5 *Microbiome-driven research

Trials should investigate whether gut microbiota composition and function (via metagenomic and metabolomic profiling) can guide personalized therapy.

#### 4.7.6 *Health economic evaluations

Prospective cost-effectiveness analyses should include clinical trials that incorporate drug costs, hospitalizations, productivity losses, and quality-of-life outcomes.

Together, these priorities aim to generate high-quality stratified evidence to inform individualized and cost-effective decision-making. Particular emphasis should be placed on multicenter RCTs testing candidate regimens (e.g., cyclic rifaximin, alone or in combination with mesalazine) in patients with higher DICA categories and on the prospective validation of biomarkers (CRP and fecal calprotectin) as treatment-selection tools—currently a key unmet need.

### 4.8 Clinical implications

Routine pharmacological prophylaxis is not currently supported for all patients after an episode of AD given the lack of consistent efficacy across randomized trials. However, an individualized risk-adapted approach should be carefully considered in selected cases. Patients with multiple previous episodes, persistent low-grade inflammation, elevated biomarkers (e.g., C-reactive protein and fecal calprotectin), or endoscopic findings such as a DICA score ≥2 may be at an increased risk of recurrence and warrant closer monitoring.

In such high-risk scenarios, the use of agents such as rifaximin or mesalazine could be explored, but only within structured clinical trials, as evidence remains inconclusive. Probiotics may have a role in symptom management when functional gastrointestinal disorders coexist but should not be used to prevent the recurrence of diverticulitis.

Until more robust data emerge, clinicians should:

– Prioritize lifestyle modifications (dietary changes, physical activity, smoking cessation, weight control) as the foundation for secondary prevention
– Use endoscopy and imaging not only to exclude malignancy but also to stratify the recurrence risk
– Avoid indiscriminate pharmacologic prophylaxis in unselected patients
– Engage in shared decision-making when considering off-label interventions
– Refer eligible patients to ongoing or future stratified trials evaluating preventive strategies

Ultimately, the implementation of tailored preventive approaches awaits confirmation from high-quality prospective studies designed around validated risk markers and standardized endpoints.

## 5 Conclusion

Despite the widespread clinical use of agents such as rifaximin, mesalazine, and probiotics, current evidence does not support their routine use for the secondary prevention of RAD. Most RCTs and meta-analyses have shown inconsistent or marginal benefits, and international guidelines remain cautious in recommending pharmacological prophylaxis.

Nonetheless, emerging data suggests that a risk-adapted approach may be valuable. Stratification tools such as the DICA score, inflammatory biomarkers (CRP and fecal calprotectin), and radiologic indicators of chronic inflammation offer promising avenues to identify patients at a higher risk of recurrence who might benefit from targeted strategies. However, none of these parameters have been prospectively validated to guide treatment selection.

This narrative review proposes an exploratory clinical framework and conceptual decision algorithm to guide future research, not just immediate clinical practice. These tools aim to foster stratified trial designs, optimize endpoint selection, and assess cost-effectiveness across risk profiles.

Until more robust, high-quality data become available, lifestyle interventions, including dietary optimization, physical activity, and smoking cessation, remain the cornerstone of the secondary prevention of RAD.
